# Effects of Poty-Potexvirus Synergism on Growth, Photosynthesis and Metabolite Status of *Nicotiana benthamiana*

**DOI:** 10.3390/v15010121

**Published:** 2022-12-30

**Authors:** Maija Pollari, Nina Sipari, Sylvain Poque, Kristiina Himanen, Kristiina Mäkinen

**Affiliations:** 1 Department of Microbiology, Viikki Plant Science Centre, University of Helsinki, 00014 Helsinki, Finland; 2 Viikki Metabolomics Unit, Organismal and Evolutionary Biology Research Programme, Viikki Plant Science Centre, University of Helsinki, 00014 Helsinki, Finland; 3 Department of Agricultural Sciences, Viikki Plant Science Centre, University of Helsinki, 00014 Helsinki, Finland; 4 National Plant Phenotyping Infrastructure, HiLIFE, Biocenter Finland, Viikki Plant Science Centre, University of Helsinki, 00014 Helsinki, Finland

**Keywords:** plant-virus interactions, viral synergism, mixed infection, phenotyping, metabolite profiling, potyvirus, potexvirus

## Abstract

Mixed virus infections threaten crop production because interactions between the host and the pathogen mix may lead to viral synergism. While individual infections by potato virus A (PVA), a potyvirus, and potato virus X (PVX), a potexvirus, can be mild, co-infection leads to synergistic enhancement of PVX and severe symptoms. We combined image-based phenotyping with metabolite analysis of single and mixed PVA and PVX infections and compared their effects on growth, photosynthesis, and metabolites in *Nicotiana benthamiana*. Viral synergism was evident in symptom severity and impaired growth in the plants. Indicative of stress, the co-infection increased leaf temperature and decreased photosynthetic parameters. In contrast, singly infected plants sustained photosynthetic activity. The host’s metabolic response differed significantly between single and mixed infections. Over 200 metabolites were differentially regulated in the mixed infection: especially defense-related metabolites and aromatic and branched-chain amino acids increased compared to the control. Changes in the levels of methionine cycle intermediates and a low S-adenosylmethionine/S-adenosylhomocysteine ratio suggested a decline in the methylation potential in co-infected plants. The decreased ratio between reduced glutathione, an important scavenger of reactive oxygen species, and its oxidized form, indicated that severe oxidative stress developed during co-infection. Based on the results, infection-associated oxidative stress is successfully controlled in the single infections but not in the synergistic infection, where activated defense pathways are not sufficient to counter the impact of the infections on plant growth.

## 1. Introduction

Simultaneous infections of two or more viral pathogens are common both in field conditions and in the wild. Although the incidence of mixed infections has not yet been thoroughly addressed, they are thought to be the norm rather than the exception [1]. Mixed infections are, for example, responsible for many well-documented and exceptionally devastating diseases in major crop plants. Examples of such diseases that have reached a pandemic scale are maize lethal necrosis disease, rice tungro disease and sweet potato virus disease [2]. In these cases, simultaneous infections stretch host plant resources to the breaking point because interactions between the pathogens enhance symptom severity and typically favor the accumulation of either virus. This phenomenon is known as viral synergism.

Poty- and potexviruses are single-stranded positive-sense RNA-viruses that infect numerous economically important crops. Poty- and potexviruses have partially overlapping host ranges, which allows co-infections that typically cause significant yield losses. Mixed infections of poty- and potexviruses were common for example in field-grown potatoes in Pakistan [3]. The documented changes in the transcriptome during poty- and potexvirus synergism as compared to single infections reveal upregulation of transcripts connected to protein synthesis and degradation, carbohydrate metabolism, responses to biotic stress, and downregulation of chloroplast functions [4]. Synergism induces the accumulation of salicylic and jasmonic acids, and increases lipid peroxidation in cellular membranes, formation of superoxide radicals in chloroplasts and dysregulation of antioxidants. Altered miRNA expression can affect symptom severity in co- infected plants. For example, expression of miRNA398 that down regulates a copper-dependent superoxide dismutase enzyme, which is a scavenger of superoxide radicals, is enhanced and may lead to reduced capacity to detoxify reactive oxygen species (ROS) generated by co-infection [5]. The synergistic interaction results in severe endoplasmic reticulum (ER) stress due to overaccumulation of the potexviral silencing suppressor protein P25, which can remodel ER membranes [6]. Once the capacity of the unfolded protein response to mitigate ER stress has been exceeded, the ER collapses with the consequence of systemic necrosis in *Nicotiana* spp.

Changes in the cell’s transcriptome and proteome during potyvirus infection are translated into changes in the metabolome [7]. A difference in the accumulation of ROS scavengers is observed in potato plants infected with either a mild N-strain of potato virus Y (PVY^N^; a potyvirus) or an aggressive PVY^NTN^ strain that causes tuber necrosis. Faster accumulation of ROS in PVY^N^ than in PVY^NTN^-infected plants probably explains the milder infection due to better balanced ROS production and their scavenging by enzymatic and non-enzymatic antioxidants. PVY multiplication associates also with significant increases in the concentrations of metabolites from phenylpropanoid pathways [7]. Our previous work revealed that the PVA silencing suppressor, helper component proteinase (HCPro), regulates the activated methionine cycle by reducing the enzymatic activity of S-adenosylmethionine synthetase [8]. This potentially interferes with the plant’s antiviral defense (reviewed in [9]). Enhanced susceptibility to PVY infection in an elevated temperature is proposed to be linked to downregulation of the temperature-dependent activities of methionine cycle enzymes in potato [10]. Increased methionine concentration and altered S-adenosylmethionine (SAM) to S-adenosylhomocysteine (SAH) ratio connect to the responses against PVY in a resistant potato cultivar [11]. The authors propose that the methionine cycle and, specifically, its transmethylation function determines resistance or susceptibility to PVY. Ectopic HCpro expression in PVX-infected plants enhances PVX subgenomic RNA expression and decreases the level of GSH, the reduced form of glutathione. The same was achieved by knocking down the gene encoding the GSH synthetase enzyme [12]. GSH is a major antioxidant of plant cells (reviewed in [13]) and therefore it’s shortage may contribute to strong oxidative stress and the severe symptoms observed during potex-potyvirus co- infection.

Model viruses in this study are potato virus A (PVA), a potyvirus [14], and potato virus X (PVX), the type member of genus *Potexvirus* [15]. PVA has a circa 10 kb genome that encodes for 11 individual proteins. Ten of the PVA proteins are processed from a single polyprotein and the 11th, P3N-PIPO, is produced by a polymerase slippage mechanism from a separate open reading frame. The PVX genome is smaller, 6.4 kb, and encodes for five viral proteins expressed from three subgenomic RNAs. Both PVA and PVX are encapsidated into flexuous filaments. While PVA is transmitted by aphids in a non-persistent manner, PVX has no known insect vector and is transmitted mainly by physical contact with infected plant parts or tools. Single infections of PVA or PVX are often mild or even asymptomatic depending on the strain, but their co-infection causes severe synergistic infection, which favors the expression of PVX RNA [12].

To provide new information for prevention and management of diseases caused by poty- and potexvirus co-infections in crop plants, we focused on determining the holistic effects of synergism on plant growth and morphology, symptoms, photosynthetic performance, and metabolite balance. We combined image-based phenotyping and metabolite analysis to compare the effects of single and mixed PVA and PVX infections in the model plant *Nicotiana benthamiana* (*N. benthamiana*).

## 2. Materials and Methods

### 2.1. Plants and Growth Conditions

*N. benthamiana* plants were germinated and grown in a greenhouse with the photoperiod set to 16 h light/8 h dark and temperature to 22 °C and 18 °C, respectively. For the phenotyping experiments seedlings (*n* = 8–9 per treatment) were transferred to the PlantScreen^TM^ phytoscope unit at the National Plant Phenotyping Infrastructure (NaPPI, University of Helsinki, Finland). The same photoperiod was maintained, but temperatures were set to 22 °C and 20 °C, respectively and relative humidity was adjusted to 60%.

### 2.2. Virus Constructs and Agroinfiltration

The PVA construct was based on the infectious cDNA (icDNA) of PVA strain B11 (GenBank accession no. AJ296311) engineered to include the red fluorescent protein (RFP) reporter between the NIb and CP cistrons [8]. PVX, the GFP-tagged PVX full-length icDNA construct was described previously [12]. As a control we used a 35S-GUS expression construct described in Eskelin et al., (2011) [16]. The PVA and 35S-GUS -constructs were transformed to *Agrobacterium tumefaciens* strain C58C1 and the PVX construct to strain GV3101.

All plants were agroinfiltrated at the 4- to 6- leaf stage. To initiate the infections, two leaves per plant were manually syringe-infiltrated with *Agrobacterium* suspension essentially as in [17]. Control plants were infiltrated with *Agrobacterium* carrying the 35S-GUS expression construct at OD_600_ 0.1. For single infections PVA or PVX *Agrobacterium* suspension at OD_600_ 0.05 was mixed with agrobacteria carrying the 35S-GUS expression construct to obtain the final OD_600_ 0.1. For the co-infection PVA and PVX *Agrobacterium* suspensions were mixed, both at OD_600_ 0.05. For imaging the agroinfiltrated plants were arranged in a randomized design. Two biologically independent repeats of the phenotyping experiment were performed.

### 2.3. Measurement of Viral Gene Expression

Virus-derived gene expression was quantified as RFP and GFP reporter fluorescence intensities representing PVA and PVX expression, respectively, as reported earlier [12]. Samples were harvested at 6 dpi from local agroinfiltrated leaves and at 10 dpi, the end point of the experiment, from systemically infected leaves. A cork borer with an internal diameter of 5 mm was used to obtain four leaf disks form near the base of systemic leaves of each plant. The 6 dpi samples were obtained from a parallel set of plants to preserve the phenotyped plants intact.

### 2.4. Image-Based Plant Phenotyping

Non-invasive whole plant imaging was performed with the PlantScreen^TM^ conveyor system at the NaPPI facility, University of Helsinki. Experimental plants were imaged prior to agroinfiltration (0 dpi) and at 3, 6, 7, 8 and 10 dpi. Side and top view images were obtained with RGB cameras GigE uEye model UI-5480SE-C/M and GigE uEye model UI-5580SE-C/M (IDS GmbH, Obersulm, Germany), respectively. Both cameras were equipped with 1/2” CMOS sensors (Aptina Imaging, San Jose, CA, USA). Plant height, leaf surface area and morphological characteristics such as compactness and eccentricity were derived from the images using the MorphoAnalysis v. 1.0.9.8. software (Photon Systems Instruments, Drásov, Czech Republic).

Thermal images were obtained with a top view mounted FLIR model A615 infrared thermal camera (FLIR, Wilsonville, OR, USA). Final images and the temperature values (minimum, maximum and average) representing the leaf surface temperatures were obtained with the PlantScreen Data Analyzer software (Photon Systems Instruments, Drásov, Czech Republic) from infrared image data.

Chlorophyll fluorescence induction was measured with a FluorCam pulse amplitude modulated system (model FC-800MF, Photon Systems Instruments, Drásov, Czech Republic) in darkness and under actinic, i.e., photosynthetically active, light. The camera shutter and sensitivity were adjusted to 20 µs and 5%, respectively. The chlorophyll imaging-based detection of virus symptoms was done based on the reduction of chlorophyll fluorescence emitted from the infected plants. Image capture was done using the quenching protocol (FluorCam 7.0 software, Photon Systems Instruments, Drásov, Czech Republic) that generated chlorophyll fluorescence parameter images and yields [18].

The plants were dark acclimated for 20 min to ensure the complete oxidation of photosystem II (PSII). In the first step plants were illuminated with a low energy light pulse that does not trigger photosynthetic light reactions and an image, representing minimum fluorescence in the dark (F_0_), was recorded. Then, a short saturating light pulse was fired to over-reduce all functional PSII complexes. Plants were immediately imaged to record the resulting maximum fluorescence (F_m_). Actinic light was then switched on to initiate photochemistry. Saturating flashes were fired and images captured after 8, 18, 28, 48 (F_m_L1-4) and 68 s (light adapted steady state, F_m_Lss) to record fluorescence during light adaptation and steady state. For the dark adaptation phase actinic light was switched off and, after a dark-relaxation step, far-red light was used to excite Photosystem I in order to completely oxidize the electron acceptor side of PSII and the plastoquinone pool. Transient fluorescence maxima were triggered by saturating light flashes (F_m_D1-4) followed by far-red light to restore the oxidated, minimum fluorescence state.

### 2.5. Chlorophyll Fluorescence Image and Data Analysis

Relevant photosynthetic parameters, including maximum PSII quantum yield (F_v_/F_m_), PSII operating efficiency (ΦPSII), non-photochemical quenching (NPQ) and the ratio of fluorescence decline (plant vitality index and measure of CO_2_ fixation [19]) were calculated for each experimental plant based on fluorescence induction kinetics.

F_v_/F_m_, a conventional and robust plant stress indicator, was chosen to score infected areas based on symptom severity [20]. The raw chlorophyll fluorescence images in .fimg format were collected and converted to .tiff format using the FIJI software according to Pavicic et al. 2021 [21]. False color images were obtained with a python script by using OpenCV [22] and PlantCV [23] packages. Script steps included: (1) binary thresholding of F_o_ tiff images to generates plant masks; (2) apply plant masks on F_v_/F_m_ tiff images; (3) multiplication of F_v_/F_m_ values by 255 to generate comparable grey scale images; (4) apply false color scale.

Masked F_v_/F_m_ tiff images of the end point of the experiment (10 dpi) were used to extract the F_v_/F_m_ value of each pixel. On these images, regions of interest (ROIs) were manually drawn over each plant using python package EasyROI from GitHub (saharshleo/easyROI: Custom ROI in Computer Vision Applications) and all pixel values were subsequently listed and saved into a .csv file. Pixel value dispersion was normalized by setting bar height sum to 100 using the python seaborn package [24]. Based on the dispersion of F_v_/F_m_ values in the healthy controls and the average F_v_/F_m_ of co-infected plants, a threshold of 0.8 was selected to score stressed and healthy pixels. This scoring threshold was used to determine the proportions of stressed leaf area in the different conditions.

### 2.6. Metabolite Extraction

Metabolites were analyzed from samples obtained from local leaves (6 dpi) and systemic leaves (10 dpi). Three replicate samples with six leaf discs were collected, snap-frozen in liquid nitrogen and stored in −80 °C prior to extraction and metabolite analysis. The experiment was replicated twice with one smaller test set of control and PVA + PVX samples. Frozen plant tissue was disrupted with a ball mill (Retch GmbH, Haan, Germany) to a fine powder and extracted twice: first with 100% methanol and then with 80% methanol while vortexing for 30 min. D_3_-methionine and D_4_-homocysteine were added as internal standards (ISTD) both at a final concentration of 25 µg/mL. The extracts were centrifuged, evaporated to dryness with a centrifugal vacuum concentrator (miVac, SP Industries, Warminster, PA, USA) and finally reconstituted in 20% methanol prior to analysis. Samples were analyzed in a randomized order. Methionine cycle metabolites were analyzed with UPLC-ESI/QTRAP/MS (Sciex, Framingham, MA, USA), while holistic metabolite analysis of specialized and semipolar metabolites was executed with UPLC-PDA-ESI/QTOF/HDMS (Synapt G2, Waters, Milford, MA, USA).

### 2.7. UPLC-QTRAP/MS Analysis of Methionine Cycle Metabolites

Targeted metabolite analysis of methionine, homocysteine, cysteine, cystathionine, SAM and SAH was performed with UPLC- 6500+ QTRAP/MS (Sciex, Framingham, MA, USA) in positive ion mode, with the multiple reaction monitoring (MRM) method. The transitions 150 → 104 for methionine, 153 → 107 for D_3_-methionine (ISTD), 136 → 90 for homocysteine, 140 → 94 for D_4_-homocysteine (ISTD), 122 → 76 for cysteine, 223 → 134 for cystathionine, 399 → 250 for SAM, and 385 → 136 for SAH were used. Ionization and transition parameters were optimized with corresponding standards with Analyst (v1.6.3) software (Table S1). Metabolites were chromatographically separated in a Waters BEH Amide column (100 × 2.1 mm, ø 1.7 µm) at 35 °C with a flow rate of 0.3 mL/min, and with elution solvents of 0.1% formic acid in both MQ water (A), and acetonitrile (B). The gradient was started with 5% A, increased to 70% at 7 min, held for 1 min, reverted to 5% A and left to stabilize for 2 min with a total analysis time of 10 min. Injection volume was set to 2 µL.

### 2.8. Metabolite Profiling with UPLC-QTOF

Non-targeted metabolite profiling of semipolar specialized metabolites was performed with UPLC-PDA-Synapt G2 QTOF/HDMS. The chromatographic separation was performed in a Waters Premier BEH C18 column (50 × 2.1 mm, ø 1.7 µm) at 40 °C connected to an Acquity UPLC instrument (Waters, Framingham, MA USA). Elution solvents were 0.1% formic acid in both MQ water (A), and acetonitrile (B) with the flow rate of 0.6 mL min^−1^. Gradient elution was started with 98% of A, linearly decreased to 88% in 3 min, then to 10% A in another 3 min, switched back to 98% at 6.01 min. With a final stabilization for 1 min the total run time was 7 min. Injection volume was 2 µL. Metabolites were analyzed in positive, sensitivity ESI-ion mode. Capillary voltage, source and desolvation temperatures, sampling and extraction cones, cone and desolvation gas flow were 3.0 kV, 120 °C, 360 °C, 30.0, 3.0, 20.0 L/h and 800.0 L/h, respectively. The mass range (*m*/*z*) was 100–700. The peak picking, integration and statistical analysis was performed with the MassLynx/MarkerLynx software (V4.2, Waters, Framingham, MA USA). Metabolites were identified by their exact molecular ion mass (*m*/*z*), their retention time order and previously published metabolite data from *N. benthamiana* (Table S3A,B).

### 2.9. Statistical Analysis of the Metabolite Data

Principal component analysis (PCA) and hierarchical clustering analysis (HCA) were performed with MetaboAnalyst (version 5.0) [25] to visualize general variation in the metabolomics data. The analyses were performed with log-transformed data with pareto scaling. False discovery rate (FDR) was applied for multiple comparisons of individual metabolite levels. *p*-value < 0.05 was considered significant and missing values were imputed with the probabilistic PCA (PPCA) method in MetaboAnalyst. Pairwise comparisons of the effects of single and mixed infections were tested with the Student’s *t*-test. Statistically significant effects (*p*-value < 0.05, FC > 1.2) on the metabolite levels were visualized with Venn diagrams [26].

## 3. Results

### 3.1. PVX-Derived Gene Expression Benefited Synergistically from a Mixed PVA-PVX Infection

**Figure 1:**
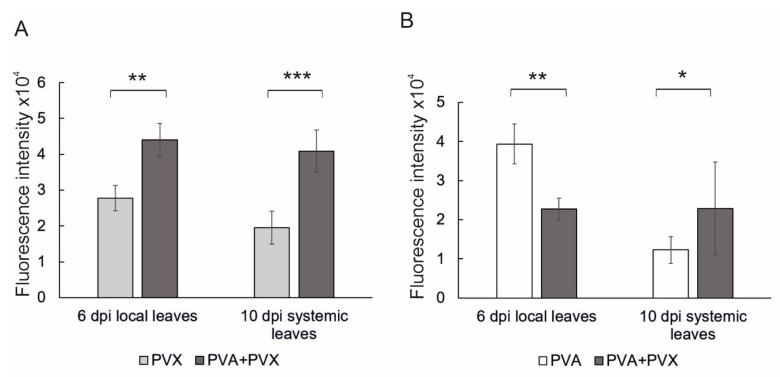
Quantification of reporter gene expression in single and mixed PVA and PVX infections revealed synergistic benefits for PVX in both local and systemic leaves. (**A**) PVX-derived GFP and (**B**) PVA-derived RFP fluorescence intensities were measured from local and systemic samples at 6 and 10 dpi, respectively. Averages of one independent experiment (*n* = 8–9 plants per treatment) are shown. Error bars indicate standard deviation and statistical significance was calculated with Student’s *t*-test (* *p* < 0.05, ** *p* < 0.01, *** *p* < 0.001).

To compare the effects of single and mixed PVA and PVX infections in *N. benthamiana*, we initiated both types of infections by agroinfiltration with strains carrying the viral icDNAs in expression vectors. Same *Agrobacterium* concentrations of the virus constructs were used in both single and mixed infections to ensure a similar initial infection pressure in the host tissue. After 6 dpi the local infection was assayed by measuring virus-derived reporter fluorescence in samples obtained from the agroinfiltrated leaves. In addition, we verified the long-distance spread and systemic presence of the viruses by measuring reporter fluorescence from young systemic leaves at 10 dpi. PVX-derived GFP expression was higher in the co-infection compared to the single infection in both local and systemic leaves, thus confirming PVA-PVX -synergism. Compared to the single infection, PVX-derived GFP expression in the co-infection was 1.6-fold higher in local leaves and 2.1-fold higher in systemic leaves (Figure 1A). In contrast, the effect of the mixed infection on PVA was asymmetric: PVA-derived RFP expression was reduced in local leaves, but the systemic infection was slightly increased (Figure 1B).

### 3.2. Effect of Single and Mixed Infections on Plant Growth, Morphology and Leaf Temperature

**Figure 2:**
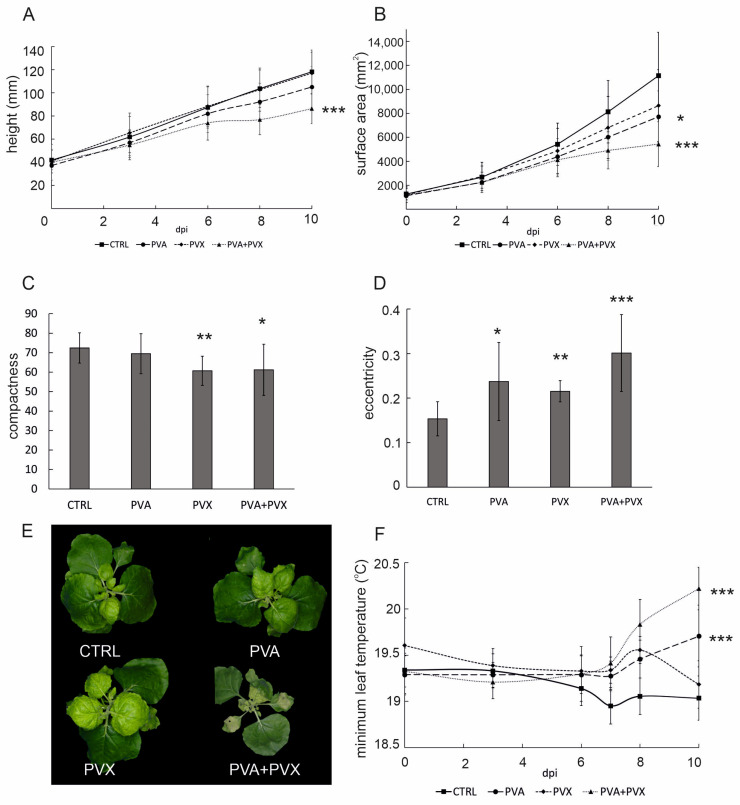
Image-based phenotyping results showing the effect of single and mixed PVA and PVX infections on (**A**) plant height, (**B**) surface area. Effects on plant morphology, i.e., (**C**) compactness and (**D**) eccentricity were calculated from image data at 10 dpi. (**E**) Representative RGB images of control, PVA, PVX and PVA + PVX -infected plants at 10 dpi. (**F**) Effect of single and mixed PVA and PVX infections on minimum leaf temperature. Plants were imaged with a thermal infrared camera. Results are presented as averages from one phenotyping experiment, error bars denote standard deviation and statistical significance was calculated by Student’s *t*-test (* *p* < 0.5, ** *p* < 0.01, *** *p* < 0.001).

In order to assess holistic effects of single and mixed PVA and PVX infections, whole plants were imaged with both top- and side-positioned RGB cameras and a top view-mounted IR camera. Growth and morphology-related characteristics, i.e., height, surface area, compactness and eccentricity were analyzed from the image data with the Morpho Analyser software. Single infections did not significantly affect *N. benthamiana* stature, but co-infected plants were 27% shorter compared to the controls (Figure 2A). Smaller size of the co-infected plants was also evident in the leaf surface area, which was reduced by circa 50% compared to the healthy control at 10 dpi (Figure 2B). Temporally, the decrease in both growth-related characteristics coincided with the systemic spread of the viruses after 6 dpi. The area of PVA-infected plants was also slightly reduced, although height remained unaffected at 10 dpi (Figure 2A,B). In contrast, the PVX single infection did not affect *N. benthamiana* height or surface area. These results reveal that young systemic leaves are especially vulnerable to the synergistic PVA + PVX infection, which markedly inhibits the growth of new leaves.

Plant morphology is the combined outcome of genetic and environmental determinants. Developmental acclimation to adverse conditions involves, for example, hormonal changes and adjustments to gene expression patterns, which collectively contribute to stress-induced morphogenic responses [27,28]. In the current setting, poty-potexvirus synergism had a significant impact not only on size (Figure 2A,B), but also on the shape of the plants. PVX and co-infected plants had a more irregular perimeter compared to the control plants and were therefore less compact (Figure 2C). Compactness indicates the distance between the leaf tip and plant center and is determined as the ratio of the surface area to the convex hull. PVX and co-infected plants had a more irregular perimeter and reduced leaf blade area compared to the control plants and were therefore less compact (Figure 2C). Single and mixed infections both resulted in the plants developing more eccentric overall shapes with PVA + PVX co-infected plants being nearly twice as elliptical as the controls (Figure 2D). Eccentricity represents the ratio of the short axis to the long axis of the plant and ranges from 0, a circle, to 1. In addition to alterations in size and shape, co-infected plants developed more striking visual symptoms than the single infections: RGB images revealed extensive chlorosis and malformation in systemic leaves and necrosis in the agroinfiltrated leaves (Figure 2E).

To connect growth, morphogenic phenotype and disease symptoms with the overall stress status of *N. benthamiana,* we used thermal infrared imaging to compare the effects of single and mixed infections on leaf surface temperature. Thermal infrared imaging is integral to plant phenotyping because leaf temperature is a sensitive stress indicator [29]. After 6 dpi minimum leaf temperature in all infections began to increase compared to the healthy control (Figure 2F), suggesting that the rate of transpiration was lowered as the virus infections spread systemically to younger leaves. Minimum leaf surface temperatures in PVA and PVA + PVX infections increased further throughout the rest of the experiment and finally were 0.7 and 1.2 °C higher than in the control, respectively. Although PVX-infected plants exhibited a similar pattern up to 8 dpi, they recovered from the initial increase in leaf surface temperature. At 10 dpi, the minimum temperature in PVX single infections had returned to the level of the healthy controls (Figure 2F).

The severe symptoms during mixed infection, stunting, altered morphology and stress as detected by increased leaf temperature, propose that several metabolic pathways were affected in these plants. As photosynthesis is a central process in determining plant energy balance, fitness and productivity, we next addressed the photosynthetic performance during single and mixed infections.

### 3.3. The Synergistic PVA + PVX Infection Reduced Photosynthetic Performance

We studied the photosynthetic performance of single and co-infected plants with chlorophyll fluorescence imaging, which allows the simultaneous measurement of photosynthetic parameters including PSII maximum and operating efficiencies, non-photochemical quenching (NPQ), and plant vitality/CO_2_-fixation (ratio of fluorescence decline).

Photosynthesis, the conversion of light energy into chemical energy takes place in photosynthetic light reactions on the chloroplast thylakoid membranes. Upon light absorption, functional PSII reaction centers engage in photochemistry. As a result, the electron acceptor side is reduced. The reaction center is said to be closed, as it cannot be further reduced. Consequently, light energy that cannot immediately be used in photochemistry, must be dissipated in a safe manner to avoid ROS production and to protect the photosynthetic apparatus. PSII chlorophyll may be de-excited via emission as heat in the process of non-photochemical quenching or as photons resulting in chlorophyll fluorescence. In dark-adapted plants PSII reaction centers are fully oxidized and yield minimum fluorescence. Because the amount of PSII chlorophyll fluorescence is inversely dependent on both the rate of photochemistry and NPQ, a high ratio of variable to maximum fluorescence (F_v_/F_m_) corresponds to a high photosynthetic rate [30]. As a fast, sensitive and non-invasive technique, chlorophyll fluorescence has become the method-of-choice in the study of photosynthetic performance in the context of plant-pathogen interactions [31].

**Figure 3:**
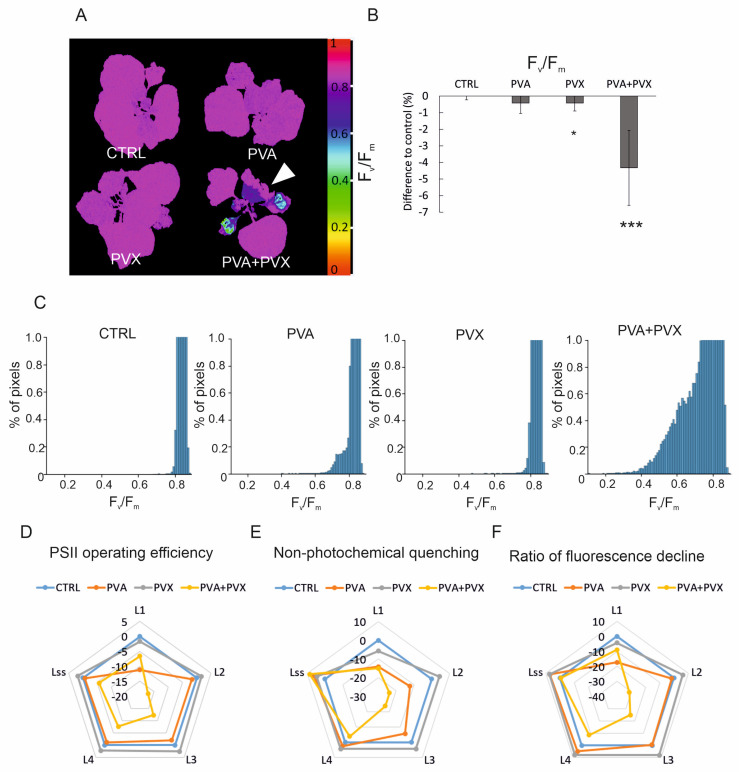
PVA + PVX co-infection lowers photosynthetic performance. (**A**) F_v_/F_m_, maximum PSII quantum yield in selected plants. A white arrow indicates a systemic leaf with low F_v_/F_m_ in the PVA + PVX co-infected plant. False color images were created by normalizing to RGB values and percentage decrease was calculated from Fluorcam whole plant image data.(**B**) Average percentage decrease in F_v_/F_m_ compared to healthy control plants (* *p* < 0.05, *** *p* < 0.001). (**C**) Dispersion of F_v_/F_m_ pixel values representing maximum PSII quantum yield in the control and single and mixed PVA and PVX infections at 10 dpi. The *X*-axis was limited to represent 1% of total counts to magnify the F_v_/F_m_ value dispersion below threshold. (**D**) PSII operating efficiency (ΦPSII), (**E**) non-photochemical quenching and (**F**) ratio of fluorescence decline during light adaptation (L1–L4) and light-adapted steady state (Lss) at 10 dpi in control, PVA, PVX and co-infected plants. Average percentage differences compared to the control are shown from one phenotyping experiment.

According to the fluorescence imaging, F_v_/F_m_, maximum PSII quantum efficiency decreased predominantly in the systemic and agroinfiltrated leaves of PVA + PVX co-infected plants (Figure 3A). The affected zone on the systemic leaves expanded and intensified from 6 to 10 dpi (Figure S1B) and matched the development of a chlorotic area in the same plants (Figure 2E and Figure S1A). The area with low PSII capacity and visible disease symptoms also showed elevated viral gene expression, which confirmed PVA-PVX synergism within this location (Figure 1). Interestingly, although viral gene expression was robust also in the systemic leaves of singly infected plants, no effect on PSII capacity was visible in the fluorescence images (Figure 3A and Figure S1B). On average, the F_v_/F_m_ of co-infected plants was decreased by 4.3% compared to the control, indicating the photosynthetic apparatus was damaged (Figure 3B). While PVA-infected plants were not significantly affected, PVX-infection caused a small but significant reduction in F_v_/F_m_. When comparing the fluorescence images to the per-plant averages, we noticed that in the co-infected plants older leaves with normal photosynthetic capacity masked the effect of viral synergism on young systemic leaves. This masking caused a smaller than anticipated decrease in the average F_v_/F_m_. Therefore, to explore the effect of the infections on photosynthetic capacity in more detail, we extracted F_v_/F_m_ values from the full plant area and plotted the dispersion of the pixel values for each treatment. The F_v_/F_m_ values of co-infected plants showed higher dispersion compared to the controls and single infections signifying a steep decline in photosynthetic capacity (Figure 3C and Figure S2). We then quantified the effects of the infections on the dispersion of F_v_/F_m_ values by counting from each plant the number of pixels with a value lower than 0.8. The threshold for these stressed pixels was chosen to represent a 5% decrease from the average F_v_/F_m_ (0.84) of healthy plants. In the PVA + PVX co-infection 22% of pixels had a F_v_/F_m_ value lower than 0.8, while the proportion of affected pixels in the control was 0.09%. Compared to the control, PVA and PVX single infections also had higher proportions of stressed pixels, 1.67% and 0.61% respectively.

Changes in chlorophyll fluorescence-based parameters reflect how well photosynthesis acclimates to light. Thus, with the fluorescence imaging, we were able to follow fluorescence induction kinetics when actinic light was switched on after darkness. Our results showed PSII operating efficiency, non-photochemical quenching, and the ratio of fluorescence decline were reduced during light acclimation mainly in PVA + PVX co-infected plants (Figure 3D–F, Table S2). Interestingly the differences compared to control were the greatest at 18 and 28 s (L2, L3) into the light period, and improved at the light-adapted steady state (68 s, Lss). In the synergistic infection at 10 dpi, PSII operating efficiency was reduced at most by 17.2%, NPQ by 23.8% and the ratio of fluorescence decline fixation by 31.3% (Figure 3D–F, Table S2). These photosynthetic parameters were significantly reduced also in the PVA single infection but only at the beginning of the light phase (8 s, L1). Because the early, 8–28 s, fluorescence decline rate was slower in the co-infected plants compared to the control, we suggest the use of light energy for photochemistry was less efficient than in the healthy plants. We observed that variation between the plants was high in the early illumination phase signifying the photosynthetic responses of the experimental plants were not uniform possibly due to individual variations in the infections (Table S2).

Photosynthesis remained mostly unchanged in the PVX single infection compared to the control (Figure 3D–F, Table S2). As an exception, PVX had a significantly increased ratio of fluorescence decline compared to the control at 48 s of light adaptation. This difference signifies possible overcompensation in CO_2_-fixation. The effect was, however, minor and was absent in the independent repeat of the phenotyping experiment.

### 3.4. Metabolite Profiles Differ between Single and Mixed Infections

To better understand the adverse effects of synergism, we explored changes in the metabolome in PVA/PVX infected *N. benthamiana* plants. We performed a non-targeted metabolite profiling analysis to analyze the broad-spectrum impact of poty-potexviral synergism compared to the individual infections. The metabolites were studied with an untargeted approach by UPLC-QTOF/MS with reversed phase (C18) separation. Multivariate statistical methods with hierarchical clustering analysis (HCA) and principal component analysis (PCA) were used to assess the overall variation in metabolite profiles of healthy controls and single and co-infected samples.

**Figure 4:**
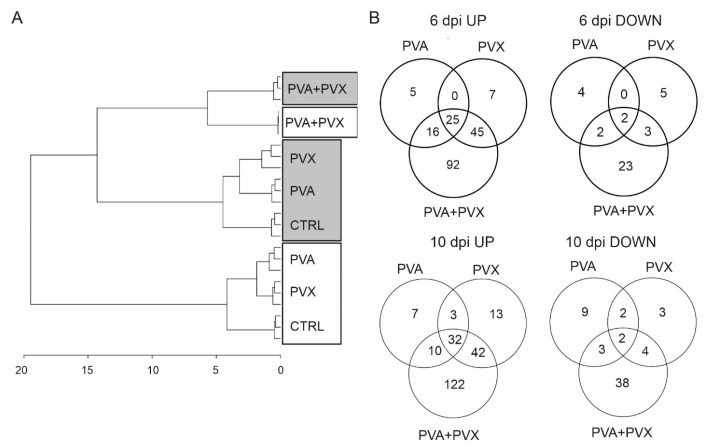
Metabolite analysis of single and mixed infections. (**A**) A dendrogram showing the differences between metabolite profiles in healthy controls and single and mixed PVA and PVX infections was generated based on hierarchical clustering analysis of the samples. 6 dpi local samples are boxed in gray and 10 dpi systemic samples are boxed in white. (**B**) Significantly up and down regulated metabolites in the infections at 6 and 10 dpi. Venn diagrams represent the numbers unique and shared metabolites in single and mixed infections compared to the control samples. Statistical significance (*p* < 0.05) was analyzed with the Student’s *t*-test and visualized with Venn diagrams.

In PCA all sample groups were clearly separated based on their metabolite profiles by the first (PC1, 42.6%) and second principal component (PC2, 22.8%) (Figure S3). The first component explained the impact of the different infections and the second PC explained the variation between local (6 dpi) and systemic leaves (10 dpi) (Figure S3). Similar clustering and variation in the metabolite profiles between systemic (10 dpi) and local (6 dpi) leaves was evident in the HCA dendrogram visualizing the distance of differences between the samples (Figure 4A). Both HCA and PCA showed clearly that the metabolite profiles of PVA + PVX co-infected plants formed their own subcluster while the other treatments grouped with respect to the sampling time and location. PVA and PVX single infections formed subclusters within the local and systemic sample groups, indicating similar metabolite profiles (Figure 4A and Figure S3)

The differences between single and mixed PVA and PVX infections compared to the healthy controls in both local (6 dpi) and systemic (10 dpi) samples were tested with the Student’s *t*-test and Venn diagrams were generated to visualize the numbers of unique and shared statistically significantly responding metabolites. The results revealed that metabolite levels mainly increased in response to the virus infections in both local and systemic leaves when compared to healthy controls (Figure 4B). 190 metabolites were significantly upregulated in the local and 229 in the systemic leaves, while 39 and 61 decreased, respectively (Figure 4B).

In systemic leaves, the levels of 32 metabolites were significantly increased in all treatments, while only two were significantly decreased. Among the 32 increased metabolites were gamma-aminobutyric acid (GABA), N-butanoylnornicotine and several phenolamides in addition to unknowns (Table S3E,F). Also, the majority of shared down-regulated metabolites were unknown. Anabasine, anabasine-n-oxide and tryptamine were exceptions that were significantly decreased in the PVA single infection and in the PVA + PVX co-infection.

Overall, the synergistic PVA + PVX infection seems to have the most significant effect: in local leaves at 6 dpi the levels of 178 metabolites increased and 30 decreased while in systemic leaves (10 dpi) 206 metabolites increased and 47 decreased (Figure 4B). Out of these changed metabolites, the majority, 52%, 77%, 59% and 81%, respectively, were limited to the synergistic infection. Taken together, the results emphasize that on the metabolic level PVA + PVX synergism induces unique and more diverse effects than either of the single infections.

### 3.5. Induction of Pathogen-Responsive and Defense-Related Metabolite Markers

The accumulation of defense-related metabolites including phenolamides, phenylpropanoids and alkaloids is a common hallmark of virus infections [32]. In the current study, many such markers were upregulated in not only the PVA + PVX co-infection but also in the single infections. Metabolites specifically emphasized in the synergistic infection included phenolamides, phenylpropanoids, pipecolic acid and nornicotine and its derivatives (Table S3A,B).

Phenolamides (grossamides, caffeoyl- and coumarylputrescine, N,N-diferuloylspermidine, and feruloylspermidine, -putrescine, -tyramine, and -tyramine glucoside) accumulated in response to all three infections (Table S3A,B). Upregulation appeared strongest in the synergistic infection: the levels of almost all detected phenolamides increased significantly in both local (6 dpi) and systemic (10 dpi) leaves of PVA + PVX co-infected plants (Table S3A,B). While the levels of some phenylpropanoids (e.g., quinic acid derivatives; caffeoyl quinic acids, coumaryl quinic acid) increased similarly in all three infections, the feruloylquinic acid and dimethylbenzoate accumulated to high levels specifically in co-infected plants (Table S3A,B). Dimethylbenzoate and feruloylquinic acid levels increased 4-fold in local leaves at 6 dpi, while their levels increased over 20-fold in systemic leaves (10 dpi), indicating more rigorous upregulation in systemic leaves.

Aromatic amino acids are required for the biosynthesis of both phenolamides and phenylpropanoids. In line with the accumulation of these specialized metabolites, we observed that the levels of the precursors, phenylalanine, tyrosine, tryptophan and corresponding amines (phenetylamine, tyramine, tryptamine, 3-methoxytyramine) increased significantly in co-infected plants (Table S3A,B). In addition, branched-chain amino acids valine and leucine, but not isoleucine, were upregulated in the co-infected plants.

The increased levels of phenolamides and their glucosylated derivatives have been previously reported to be associated with H_2_O_2_ induction and signaling during the hypersensitive response as well as antioxidative and toxic properties against pathogens [33,34]. On the other hand, their levels decrease during senescence in *Nicotiana tabacum* [35]. In addition to the marked enhancement of phenolamides and phenylpropanoids, the levels of pipecolic acid were over 20-fold higher in the co-infection compared to the control in both local and systemic leaves (Table S3A,B). Pipecolic acid belongs to a group of known senescence markers in plants together with aromatic and branched-chain amino acids [36,37,38]. Moreover, it has been reported to act as an endogenous regulator of inducible plant immunity [39].

### 3.6. The Methionine Cycle and Glutathione Metabolism Are Imbalanced in the Synergistic Infection

In plants, sulfate absorbed from the soil is reduced to cysteine, which is directed to the synthesis of sulfur-containing compounds including methionine. The methionine cycle is a sensitive target of poty-potexviral infections and presents a potential target for resistance modifications [9,12]. Therefore, to gain a more thorough understanding of the responses of the methionine cycle to single and mixed poty- and potexvirus infections, we focused on examining its intermediates in more detail. In contrast to the holistic approach, methionine cycle intermediates from *N. benthamiana* were analyzed with a targeted approach by UPLC-QTRAP/MS.

**Figure 5:**
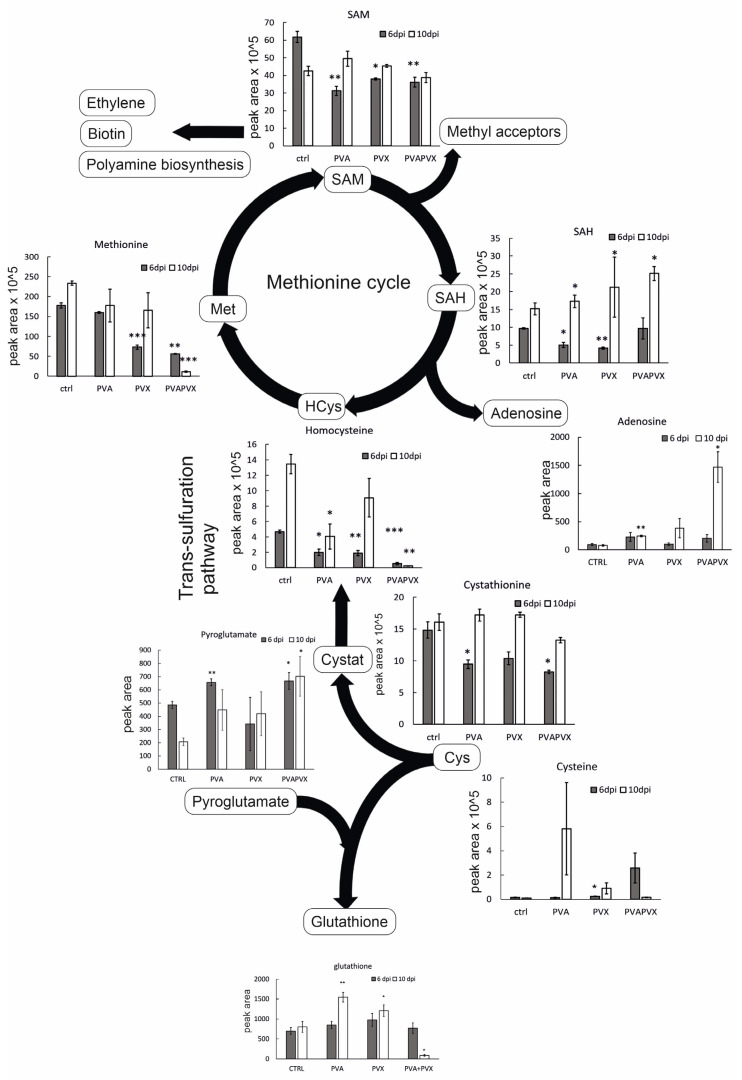
Levels of methionine cycle intermediates and selected metabolites in single and mixed infections. Data represents averages of one experiment, statistical significance compared to the healthy controls of the same day was calculated with Student’s *t*-test (* *p* < 0.5, ** *p* < 0.01, *** *p* < 0.001). The following abbreviations are used: cysteine (Cys), cystathionine (Cystat), homocysteine (HCys), methionine (Met), S-adenosylmethionine (SAM), S-adenosylhomocysteine (SAH). The diagram was created with BioRender.

Based on the analysis, the levels of most of the intermediates of the methionine cycle decreased in response to the single and mixed infections suggesting a general repression of methionine synthesis and the methionine cycle (Figure 5). While there was no lack of cysteine in single or mixed infections, a significant increase was detected in PVA-infected plants at 10 dpi (Figure 5). This suggests that the initial stages of sulfur assimilation or the generation of cysteine were not impaired by the infections. Cysteine is converted via cystathionine into homocysteine, which serves as a source for de-novo methionine synthesis in plastids. Homocysteine thus connects the chloroplastic sulfur-assimilation path and the cytoplasmic methionine cycle (reviewed in [40]). The level of homocysteine decreased by 98% in the PVA + PVX co-infection in both local and systemic leaves (Figure 5). The effect was synergistic, as the decrease was greater than in the single infections. Supply of cystathionine, the precursor of homocysteine, could be the limiting factor at 6 dpi as its level was significantly reduced in local leaves at 6 dpi. In contrast, unchanged levels of cystathionine in the systemic leaves imply that other factors than a limited supply of the precursor are responsible for reduced homocysteine levels in the PVA + PVX co-infection at 10 dpi (Figure 5).

Methionine content was significantly decreased in PVA + PVX co-infected local and systemic leaves (Figure 5). In these samples methionine levels were downregulated by circa 70 and 95%, respectively, compared to the control. Likewise, the PVX infection alone decreased the level of methionine in local leaves (Figure 5). SAM, which is synthesized from methionine by S-adenosyl methionine synthetase, was similarly decreased in all infections at 6 dpi (Figure 5). In contrast, SAM content did not change in the systemic leaves at 10 dpi (Figure 5). SAM is the universal methyl group donor in cellular transmethylation reactions that produce methylated target substrates and SAH, which regulates the process by inhibiting methyltransferases (reviewed in [41]). In our experiments, SAH levels decreased in the single infections in local leaves but increased in all infections in the systemic leaves (Figure 5).

**Figure 6:**
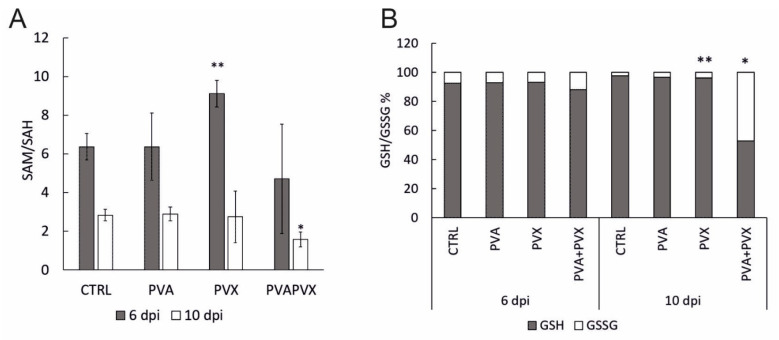
(**A**) SAM/SAH ratio and (**B**) glutathione balance in local (6 dpi) and systemic (10 dpi) leaves of healthy controls, PVA and PVX single infections and the PVA + PVX co-infection. Results are shown from one independent experiment, error bars in A) represent standard deviation and statistical significance was calculated using Student’s *t*-test (* *p* < 0.5, ** *p* < 0.01).

Since SAH, like SAM, is an important feedback regulator of the methionine cycle, the SAM/SAH ratio represents the overall methylation potential of the plant more accurately than the individual levels of the metabolites [42]. Younger leaves appear have a lower methylation potential compared to older leaves as the SAM/SAH ratio was lower in the systemic leaves compared to the local leaves (Figure 6A). In the local leaves, the SAM/SAH ratio in PVA and PVA + PVX infections was similar to the control while the PVX-infected plants had a higher ratio (Figure 6A). In the systemic leaves, however, the SAM/SAH ratio was significantly lower in the PVA + PVX co-infection compared to the control while both single infections maintained a normal ratio (Figure 6A). The reduced SAM/SAH ratio in the co-infection indicates that PVA-PVX synergism impairs the plant’s transmethylation potential. The condition may lead to the hypomethylation of DNA and RNA and thus have consequences for the regulation of gene expression.

SAH hydrolysis by SAH hydrolase produces adenosine and regenerates homocysteine. Interestingly, adenosine accumulated 19-fold higher in the systemic leaves of PVA + PVX co-infected plants than in the control (Figure 6). Adenosine levels were significantly higher also in the PVA single infection compared to the control. The accumulation of adenosine in the PVA + PVX co-infection suggests a disruption in either SAH hydrolase activity or in downstream phosphorylation of adenosine to adenosine monophosphate. Excess adenosine inhibits SAH hydrolase, which probably contributed to the accumulation of SAH in the systemic samples (Figure 5).

Glutathione, the γ-glutamyl-cysteinyl-glycine tripeptide, is an important antioxidant that mitigates stress in plants by neutralizing toxic oxidative compounds. We found that total glutathione was unaffected in local leaves at 6 dpi, but its level in the systemic leaves increased significantly in response to the PVA and PVX single infections. The co-infection, in contrast, decreased total glutathione levels by 93% (Figure 5).

Since the cellular glutathione pool balances reduced (GSH) and oxidized (GSSG) forms of glutathione to optimize the redox environment, cells maintain a high percentage of GSH ready at hand for the elimination of ROS [43,44]. Consequently, an increased proportion of GSSG indicates oxidative stress. Our results showed that in healthy controls and single infections the proportion of GSH was over 90%, while the PVA + PVX co-infection reduced it to 53% in systemic leaves (Figure 6B). In addition, the level of GSSG in the co-infection was 19-fold higher than in the control and its share of the total glutathione pool increased from 3% to 47%. There was also a small but significant increase in PVX-infected systemic leaves (Figure 6B).

These results reveal that both single and mixed infections challenge the redox status of the cells. We found the PVA + PVX co-infection caused stronger oxidative stress than the single infections and the cellular glutathione balance reflected the severity of the stress. Plants with single infections were able to increase the production of GSH to maintain the optimal GSH/GSSG ratio and, therefore, were able to alleviate the effects of oxidative stress. Poty-potexviral synergism, however, overloaded the system and the plants were unable to maintain the volume of the total glutathione pool. Furthermore, in the PVA + PVX co-infection, a serious imbalance of the GSH/GSSG ratio suggested extensive oxidative stress.

## 4. Discussions

In agriculture synergistic infections may destroy entire harvests, as happened in Kenya in 2012 during a maize lethal necrosis disease epidemic caused by co-infection of a potyvirus (sugarcane mosaic virus) and a machlomovirus (maize chlorotic mottle virus) [45,46]. Poty- and potexvirus co-infections are also estimated to cause significant, 40–80%, reductions in yield [3,47]. PVX and two potyviruses (PVY and plum pox virus) are ranked on the top ten list of most studied and economically damaging plant viruses with PVY being the most important potyvirus [48,49]. With many crops qualifying as hosts for both poty- and potexviruses, their synergism presents high risks for global food security. Furthermore, accelerating climate change may alter not only the populations and activity of insect vectors transmitting viruses, but also weaken plant antiviral defenses [50,51,52]. 

The main body of our knowledge of plant virus-host interactions comes from the investigation of isolated pathosystems. Consequently, considerably less is known about the impact of overlapping infections although solutions to prevent and control the negative impact of these mixed infections are urgently needed. Therefore, to enable the development of resistance and protective measures, more research is required into the unique effects of synergistic infections and the underlying molecular mechanisms.

Here we combined automated imaging with metabolite analysis to establish a comprehensive overview of plant well-being and responses during single and mixed PVA and PVX infections. According to Castro-Moretti et al., (2020) [53], the metabolite perspective is a vital next step for understanding plant-pathogen interactions and for developing sustainable resistance. Our approach of conceptual integration, i.e., separate analysis of data sets followed by comparison and matching [54], allowed the formation of relevant links between key processes and metabolic pathways that contribute to poty-potexviral synergism. While the phenotyping system enabled efficient time-course analysis of growth, leaf temperature stress and photosynthesis, metabolic analyses required invasive sampling and were thus restricted to chosen time points and tissues. Plant metabolites are highly diverse and their fluxes dynamic, so the available method presents quite a limited snapshot of the metabolic landscape in infected plants. Recently Hall et al. [55] overviewed the challenges of combining plant phenotyping and metabolomics. Despite the challenges, they envisioned the implementation of novel methods that would allow high-throughput metabolite analysis in parallel with phenotyping in the future [55]. Not only are there obstacles in developing metabolomics platforms, but also in combining information from different omics levels (genomic, transcriptomic, proteomic, metabolomic and phenomic). Such approaches hold potential for deepening our understanding of the dynamics of biological systems. There is, therefore, an urgent need to develop approaches towards integration of multiple omics level information [56]. In the study of plant-pathogen interactions first steps in this direction have already been taken [57,58,59,60,61].

Results of this study showed diminished growth and enhanced symptoms in co-infected plants, which aligned with previous reports on the negative effects of poty-potexviral synergism [3,62,63]. Moreover, our results revealed the systemic spread of the PVA-PVX co-infection led to stress-induced morphogenic responses, increased leaf temperature and a decline in photosynthetic capacity. In addition, the synergistic infection disrupted both the methionine cycle and glutathione balance, which reduced the plant’s ability to alleviate oxidative stress. Co-infected plants invested resources into a more diverse array of defense related metabolites than plants with either of the single infections.

Leaf temperature depends mainly on the rate of water evaporation through small pores called stomata and is governed by stomatal responses to environmental cues [64]. Plants usually close their stomata to conserve water through reduced transpiration, but the tradeoff is lowered photosynthesis due to reduced CO_2_ flux. Short-term stomatal responses involve guard cell movement to adjust stomatal aperture while long-term drought or other stress conditions may reduce the density of stomata [65,66]. Thus, the increased leaf temperatures observed in PVA and PVA + PVX infections can be either due to closed stomata or to the development of fewer stomata in the systemic leaves. Virus infections can affect both the number and aperture of stomata on the leaves of diseased plants. For example the tobamoviruses tobacco mosaic virus (TMV) and turnip vein clearing virus lowered the stomatal index (number stomata/(number of epidermal cells + stomata)) by circa 10%, respectively, in *Nicotiana tabacum* and *Arabidopsis thaliana* [67]. Short-term stomatal responses usually take place within minutes, so the long-lasting reduction in leaf surface temperature in the PVA and PVA + PVX infections implies a more permanent infection-associated reduction in stomatal aperture or density. Turnip mosaic virus (TuMV, genus *Potyvirus*) infection in *Arabidopsis thaliana* induced stomatal closure [68]. Parallel to our results with PVA and the PVA + PVX co-infection, Manacorda et al. (2021) [68] showed the TuMV infection increased leaf temperature from 8 dpi onwards, which supports the impact of the systemic infection in symptom manifestation. Enhanced accumulation and altered regulation of abscisic acid (ABA) signaling appears to be involved in the stomatal closure triggered by the TuMV infection [68]. Although ABA was not included in the metabolite profile in this study, we instead found increased levels of the signaling molecule GABA in both local and systemic leaves of co-infected plants. GABA is involved in the regulation of various pathways promoting tolerance to stresses and it affects stomatal closure by enhancing ABA production [69,70,71,72]. While stomatal closure improves drought tolerance and ABA has been shown to improve plant defenses against another potyvirus, plum pox virus [73], prolonged closure of stomata eventually has a negative impact on plant growth. 

Chloroplast-derived ROS and salicylic acid (SA) play a pivotal role in establishing effective plant immunity. A successful defense response via the production of these molecules leads to hypersensitive response (HR), a form of programmed cell death (PCD), and confinement of the pathogen to the necrotic lesion [74,75,76]. A recent study indicated that the increase in chloroplast redox state at the cell death zone is linked to PCD signaling in a SA independent manner whereas the signaling for HR-conferred resistance to the virus is SA-dependent [77]. In susceptible infection, confinement of the virus/viruses is not successful. Nevertheless, SA level increases due to potyvirus infection [78]. Based on our results the specific substantial increase in pipecolic acid proposes that signaling for systemic acquired resistance was induced. It appears the activation of systemic acquired resistance pathways was inadequate to contain the viruses as robust systemic infection develops in the PVA + PVX co-infection in *N. benthamiana.*

While ROS are important signaling molecules of immune reactions, excess ROS damage macromolecules including photosynthetic complexes [75]. Oxidative stress management strategies are essential for the upkeep of the chloroplast redox state and active photosynthesis and have been well-characterized from the perspectives of different abiotic stresses (reviewed in [79]).

In the current study extensive oxidative damage to the photosynthetic apparatus in the systemic leaves was mirrored by decreases in key photosynthetic parameters (F_v_/F_m_, PSII operating efficiency, NPQ and ratio of fluorescence decline). The overall decline in photosynthesis observed in the co-infected plants could be due to more extensive damage to PSII, slower repair of PSII or lower photosynthetic electron transport rate. Young *N. benthamiana and N. tabacum* leaves are vulnerable to tobamovirus infection-induced stress, which has been reported as a decline in several photosynthetic parameters in pepper mild mosaic virus and TMV infections, respectively [80,81]. *N. benthamiana* NPQ responded especially sensitively to a pepper mild mottle virus infection the during the early light adaptation phase [82].

Because many previous studies have highlighted the negative impact of infections on photosynthesis, we were surprised the single PVA and PVX infections had very mild or no effect on host photosynthetic parameters. In many cases virus infections tend to decrease the expression of photosynthesis-related genes and proteins [83,84]. One reason for the apparent lack of impact on photosynthesis could be due to the fact that the sampling for transcriptomic and proteomic studies is often restricted to the systemically infected leaves, whereas our results reflect the overall status of whole plants. Another explanation could be the time range of the experiment: it is possible that the effects of the single infections were not yet detectable by 10 dpi. For example, the potyvirus sweet potato feathery mottle virus infection in sweet potato reduced photosynthesis predominantly in the latter stage of the infection (13–29 dpi) while the effects of single infections on photosynthesis were not evident in the early infection [85]. In the case of PVA and PVX we therefore would expect the single infections to eventually reduce photosynthetic activity, as the negative effects of the infections accumulate over time.

Based on our results, host photosynthesis in the single PVA and PVX infections was efficiently maintained by a successful upregulation of antioxidative responses. Although the spread of the viruses was not limited, as would happen in the HR, the symptoms of the respective infections were mild. It seems that cells avoided damage to the photosynthetic apparatus by keeping oxidative stress under control for example by increasing the cellular glutathione pool. In contrast, in the synergistic infection, young systemic leaves were unable to rally enough GSH to manage the stress burden and therefore exhibited the severest reduction in photosynthetic capacity. After 6 dpi we also observed chlorosis in the systemic leaves of co-infected plants, where photosynthetic capacity was correspondingly low. The size of the light harvesting antenna of PSII is a target for regulation in adverse conditions with a high risk of chloroplast-derived ROS production due to overexcitation of PSII [79]. The yellowing may thus reflect a down-regulation of chlorophyll binding proteins, which was shown by García-Marcos et al. [4] on transcript level in the PVY-PVX co-infection (data accessible at NCBI GEO database [86], accession GSE15538). 

The glutathione synthesis pathway, during which cysteine, glutamate and glycine are joined together in a tightly regulated process in the cytoplasm, is connected to a pathway that is initiated at chloroplasts and leads to the cytoplasmic methionine cycle. One branch of this pathway leads to ethylene synthesis (pathway reviewed in [87]). Sulfate absorbed from the soil is reduced to cysteine, which is directed to the synthesis of sulfur-containing compounds including methionine. Methionine synthesized de novo in chloroplasts is transported to the cytoplasm where it serves not only as a proteinogenic amino acid, but also as a precursor for SAM (also called AdoMet) production. In the process of donating the methyl group in cellular methyl transferase catalyzed reactions, SAM is converted to SAH, which is further processed in the activated methionine cycle to homocysteine and recycled back to methionine. In addition to its function as a methyl donor, SAM serves as a precursor for polyamines and biosynthetic pathways manufacturing the phytohormone ethylene. The latter requires the conversion of SAM to the ethylene precursor 1-aminocyclopropane-1-carboxylic acid (AAC) and 5-methylthioadenosine (5-MTA). 5-MTA proceeds to the Yang cycle for methionine salvation in a reaction that regenerates adenine and methionine. When methionine accumulation in cells becomes insufficient, activation of the transsulfuration pathway, running from cysteine through cystathionine to homocysteine, still offers an opportunity to restore a sufficient methionine levels. 

Remarkably, metabolite analysis of PVA + PVX co-infected plants indicates that the maintenance of normal glutathione, homocysteine and methionine levels is not successful in the systemically infected leaves. The accumulation levels of these metabolites remain low although the precursor levels, cystathionine for homocysteine production via transsulfuration and pyroglutamate and cysteine for glutathione biosynthesis, are present at non-altered concentrations. This may indicate that cysteine is directed more strongly towards the transsulfuration pathway than to glutathione synthesis. Nevertheless, it appears that the demand for methionine exceeds the cell’s capacity to restore normal levels through homocysteine and the methionine salvage pathway. Low methionine and SAM levels influence the physiology of both the cells and the whole organism, leading to transcriptional and signaling states that remodel metabolic programs with the aim of maintaining methionine metabolism [88].

TuMV infection alone more than doubles ethylene accumulation [78]. Our metabolic analysis did not include the detection of volatile ethylene. Instead, a metabolic shift towards high accumulation of polyamines like spermidine-derivatives from SAM was observed.

SAH hydrolase is an enzyme responsible for cellular adenosine accumulation. The abnormally strong accumulation of SAH and adenosine indicates that some of the enzymatic reactions relevant for balancing the methionine cycle have been inhibited under the prevailing conditions. The inhibition of SAH hydrolase during PVA + PVX co-infection was hypothesized in De et al., 2018 [12] and reinforced by this study. The interactions of the potyviral HCPro with SAM synthetase and SAH hydrolase [8] may contribute to increasing the amount of SAH, reducing methylation potential, and by reducing the methylation-linked stabilization of sRNAs to suppress antiviral RNA silencing. High accumulation of adenosine to the extracellular space increases the susceptibility of *Arabidopsis* plants to a necrotrophic fungus *Botrytis cinerea* and reduces photosynthetic capacity [89]. On the other hand, an adenosine analog cordycepin is a molecule with antiviral, antifungal, antibacterial, and many other pharmacological activities [90]. Cordycepin, 3’deoxy-adenosine, competes with adenosine for binding to adenosine receptors. It is an inhibitor against SARS-CoV-2 replication in human cells [90]. An interesting question for future studies is whether the high accumulation of adenosine serves in defending *N. benthamiana* against the PVA or PVX accumulation or could it have a role in advancing the severe disease through reduced photosynthesis as in the case of *Botrytis cinerea*.

As expected, defense-related specialized compounds dominated the metabolite landscape in the synergistic infection in *N. benthamiana*. This observation agreed with previous work where, for example, tobacco plants have been shown to employ polyamines as part of the TMV-induced HR [91,92]. Phenylpropanoids also accumulate in response to several viruses including PVY [7,93,94,95]. There seems to be a range of defensive roles for these compounds. For example, the activity of phenylalanine ammonia lyase, a key enzyme in the phenylpropanoid biosynthesis pathway, correlated positively with plant resistance to aphids [96,97]. This could be possible also in the context of poty-potexviral synergism, as phenylalanine ammonia lyase transcripts were upregulated in the PVY + PVX co-infection ([4], data accessible at NCBI GEO database [86], accession GSE15538). Furthermore, pipecolic acid, a non-proteinogenic amino acid that accumulated intensively in the synergistic infection, has been reported to act as an endogenous regulator of inducible plant immunity [39]. Because the untargeted approach was conducted in positive ion mode, we were not able to detect jasmonic acid, an important plant stress hormone and SA antagonist, in this study. The inclusion of jasmonic acid and jasmonic acid-isoleucine would be of interest as part of a future study but would require a targeted approach employing multiple reaction monitoring with a triple quadrupole MS instrument.

The accumulation of many defense-related metabolites was strongest in the systemic leaves of PVA + PVX co-infected plants indicating that antiviral responses were still operating at high capacity. Yet the response was not enough to counter the infection, as growth had declined severely. In this situation the allocation of already scarce resources to the high-cost synthesis of specialized metabolites appears futile. Why do co-infected plants direct energy to the synthesis of specialized metabolites even though their already elevated presence is clearly not sufficiently effective against the infection? This question could be one starting point for continued research into dissecting the complex molecular foundations of synergistic poty-potexvirus infections.

In conclusion, our results provide evidence for the devastating impact of uncontrolled oxidative stress in a synergistic poty-potexvirus infection. Comparison of the single and mixed PVA and PVX infections demonstrated that the activation of defense and oxidative stress-related pathways was successful in the single infections where the host plants maintain homeostasis. In contrast, stress caused by the synergistic infection was so severe the host’s defenses, although strongly activated, were not adequate to uphold photosynthetic activity and growth. Future directions arising from our study include possibilities for exploring the unknown metabolites to discover candidate antiviral compounds that could be utilized in safe and sustainable plant protection. The cysteine precursor L-2-oxo-4-thiazolidine-carboxylic acid and the flavonol quercetin are current examples of control agents with the potential to support the healthy growth of virus-infected plants by alleviating oxidative stress [98,99].

Artificial intelligence and deep learning strategies are already indispensable for generating meta-level networks of omics information. Future systems-scale research on plant-virus interactions and defense responses will benefit from advances in the integration of data from multiple omics levels. [55,56]. Combined with sufficient background information about the relationship between phenotype and metabolome, applications such as the tailoring of biosynthetic pathways by CRISPR-Cas9 [100] may pave the way for priming the plant metabolite profile for antiviral defense.

## Supplementary Materials

The following supporting information can be downloaded at: https://www.mdpi.com/article/10.3390/v15010121/s1, Figure S1: Representative images showing the time-scale comparison of growth and development and maximum PSII quantum yield in control, PVA, PVX and co-infected plants; Figure S2: Full dispersion of F_v_/F_m_ pixel values; Figure S3: PCA score plot of differentially regulated metabolites from all control, PVA, PVX and co-infected samples; Table S1: Ionization and transition parameters for UPLC- 6500+ QTRAP/MS analysis of methionine cycle metabolites; Table S2: Effect of single and mixed PVA and PVX infections on selected photosynthetic parameters during light adaptation (L1-4) and light-adapted steady state (Lss); Table S3: Levels of metabolites detected by UPLC-MS and statistically significant metabolites in local and systemic leaves and partial least squares discriminant analysis (plsda) loadings [33,34,35,101,102,103,104,105,106,107].
