# Supplementary material for: Effects of Poty-Potexvirus Synergism on Growth, Photosynthesis and Metabolite Status of Nicotiana benthamiana
Source: Viruses. 2022 Dec 30;15(1):121. doi: 10.3390/v15010121 (PMC9867248; doi:10.3390/v15010121)
Supplement: Supplementary file 1 [file viruses-15-00121-s001.zip › viruses-2097447-supplementary/Table S1.pdf]

Table S1 Ionization and transition parameters for UPLC- 6500+ QTRAP/MS analysis of methionine cycle metabolites

DECLUSTERING POTENTIAL (DP)	ENTRANCE POTENTIAL (EP)	CURTAIN GAS	COLLISION GAS	ION SPRAY VOLTAGE	TEMPERATURE ^{o}C	ION SOURCE GAS 1	ION SOURCE GAS 2
**5.0**	10.0	20.0	medium	4500	450	10.0	10.0
