# Supplementary material for: Effects of Poty-Potexvirus Synergism on Growth, Photosynthesis and Metabolite Status of Nicotiana benthamiana
Source: Viruses. 2022 Dec 30;15(1):121. doi: 10.3390/v15010121 (PMC9867248; doi:10.3390/v15010121)
Supplement: Supplementary file 1 [file viruses-15-00121-s001.zip › viruses-2097447-supplementary/Table S2.pdf]

Table S2 Effect of single and mixed PVA and PVX infections on selected photosynthetic parameters during light adaptation (L1-4) and light-adapted steady state (Lss) at the end point of the experiment, 10 dpi. ΦPSII = Photosystem II operating efficiency, NPQ = non-photochemical quenching, Rfd = Ratio of fluorescence decline, plant vitality factor/CO_2 fixation. Results are averages of one phenotyping experiment, sd signifies standard deviation and Student's t-test was used to calculate statistical significance (NS= not significant, * = P<0.05, ** = P<0.01, *** P<0.001).

Light adaptation		***L1, 8 s***				***L2, 18 s***				***L3, 28 s***				***L4, 48 s***				***Lss, 68 s***			
PSII parameter	treatment	% difference to control	sd	t-test and significance		% difference to control	sd	t-test and significance		% difference to control	sd	t-test and significance		% difference to control	sd	t-test and significance		% difference to control	sd	t-test and significance	
ΦPSII	CTRL	0,0	3,8			0,0	4,9			0,0	2,2			0,0	3,3			0,0	3,4		
	PVA	-11,2	8,4	6,8E-03	**	-1,7	8,6	6,2E-01	NS	-2,0	4,8	3,0E-01	NS	-1,1	3,8	5,2E-01	NS	-0,6	3,3	7,0E-01	NS
	PVX	-1,7	4,5	4,2E-01	NS	1,6	4,2	4,9E-01	NS	2,5	3,4	9,5E-02	NS	2,2	5,0	2,1E-01	NS	1,9	3,2	2,5E-01	NS
	PVA+PVX	-6,6	7,3	3,4E-02	*	-17,2	3,6	4,5E-07	***	-12,3	4,2	4,7E-06	***	-7,7	3,7	1,9E-03	**	-5,7	5,3	1,7E-02	*
NPQ	CTRL	0,0	8,7			0,0	8,9			0,0	7,3			0,0	7,0			0,0	8,5		
	PVA	-14,0	6,6	1,9E-03	**	-12,2	7,6	8,0E-03	**	-5,7	7,8	1,4E-01	NS	2,4	7,0	4,9E-01	NS	6,3	13,7	2,8E-01	NS
	PVX	-5,7	8,0	1,8E-01	NS	4,3	9,8	3,6E-01	NS	4,3	8,3	2,8E-01	NS	4,2	9,1	3,1E-01	NS	4,6	16,1	4,8E-01	NS
	PVA+PVX	-15,0	7,9	1,4E-03	**	-23,8	11,8	2,2E-04	***	-23,8	12,9	3,6E-04	***	-3,9	10,4	3,7E-01	NS	8,6	7,2	3,5E-02	*
Rfd	CTRL	0,0	10,2			0,0	5,6			0,0	4,6			0,0	6,5			0,0	7,5		
	PVA	-17,3	14,1	1,3E-02	*	-1,6	15,8	7,9E-01	NS	-0,4	10,9	9,3E-01	NS	4,9	7,1	1,6E-01	NS	7,0	7,7	7,7E-02	NS
	PVX	-4,4	14,3	4,9E-01	NS	6,1	12,5	2,3E-01	NS	8,0	11,9	1,1E-01	NS	7,8	6,9	3,1E-02	*	7,4	7,4	5,8E-02	NS
	PVA+PVX	-8,9	14,9	1,6E-01	NS	-31,3	5,9	3,6E-09	***	-24,9	11,4	9,2E-05	***	-8,3	11,9	9,0E-02	NS	-0,5	10,9	9,1E-01	NS
